# Efficient right ventricular shape modeling using a dual active shape model

**DOI:** 10.1186/1532-429X-18-S1-W26

**Published:** 2016-01-27

**Authors:** Hossam El-Rewaidy, El-Sayed H Ibrahim, Ahmed S Fahmy

**Affiliations:** 1 University of Michigan, Ann Arbor, MI USA; 2 Nile University, Cairo, Egypt

## Background

Active shape models (ASM) showed to have potential for segmenting the right ventricle (RV) in CMR images. Nevertheless, the large variability and complexity of the RV shape do not allow for concisely capturing all possible shape variations among different patients and anatomical cross-sections. Noticeably, the latter increases the number of iterations required to converge to a proper solution and reduces the segmentation accuracy.

## Methods

**Figure 1:**
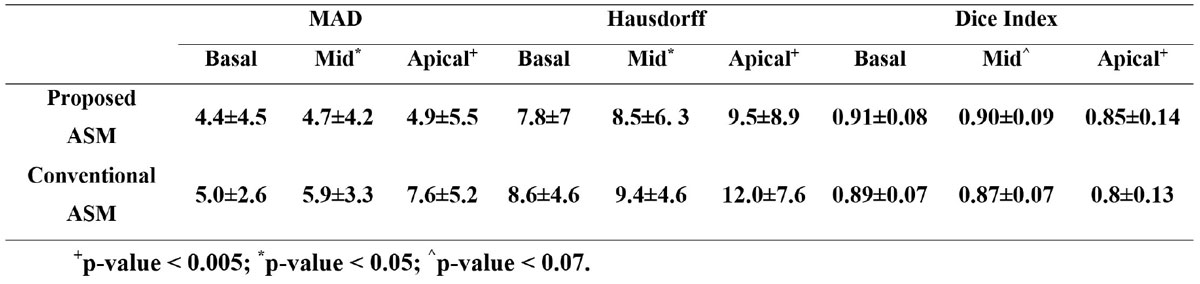
**Mean ± SD of the mean absolute distance (MAD), Hassdorff, and Dice Index measures of the segmented contours at basal, mid-cavity, and apical levels using the proposed double-ASM and conventional ASM methods with respect to ground truth manual segmentation**.

In this work, a new ASM framework has been developed for efficiently modeling the RV shape in short-axis CMR images. In this framework, the RV contour is split into two simpler segments, septal and free wall, whose shape variations are independently modeled using two separate (dual) ASM models Figure 1(a). The contour splitting is done at the location of the RV insertion points into the septal wall. Further, instead of using the conventional Procrustes method, the RV contours are aligned using the Bookstein coordinate transformation, which uses the RV insertion points as landmarks to nonlinearly align the RV contours (Figure 1(b)). The proposed framework allows for building one ASM model from all short-axis slices. The developed technique has been tested on a dataset from 10 patients imaged with cine CMR.

## Results

Table 1 shows the mean ± SD errors between the contours produced by the proposed and conventional ASM models with respect to manually delineated contours at the three different cross-sectional slices. As can be seen in Table 1, the performance of the proposed ASM framework is better than that of the conventional ASM model. This is evident by the lower value of the Mean Absolute Distance (MAD) and Hausdorf measures and higher value of the Dice index. Figure 1(c) shows the evolution of the ASM models from the initial contour to the contours at iterations number 5 and 20 for two patients. It can be seen in the figure that the initial contour of the proposed ASM framework is much better than that of the conventional ASM model. The figure also shows that the proposed ASM framework converges after almost 5 iterations whereas the conventional ASM model needs 15-20 iterations to correctly delineate the RV contour. The average computation times for segmenting one slice using a personal computer were 0.09 s and 0.17 s for the conventional and proposed ASM models, respectively. Nevertheless, the parallelized nature of the problem renders this difference insignificant.

## Conclusions

The developed double-ASM RV segmentation technique outperforms the conventional ASM framework and can efficiently model complex RV shape variation with more accuracy in less iteration steps. Although the proposed framework extracts only the RV endocardium, the epicardium can be segmented through dilating the endocardium contour.

**Figure 2:**
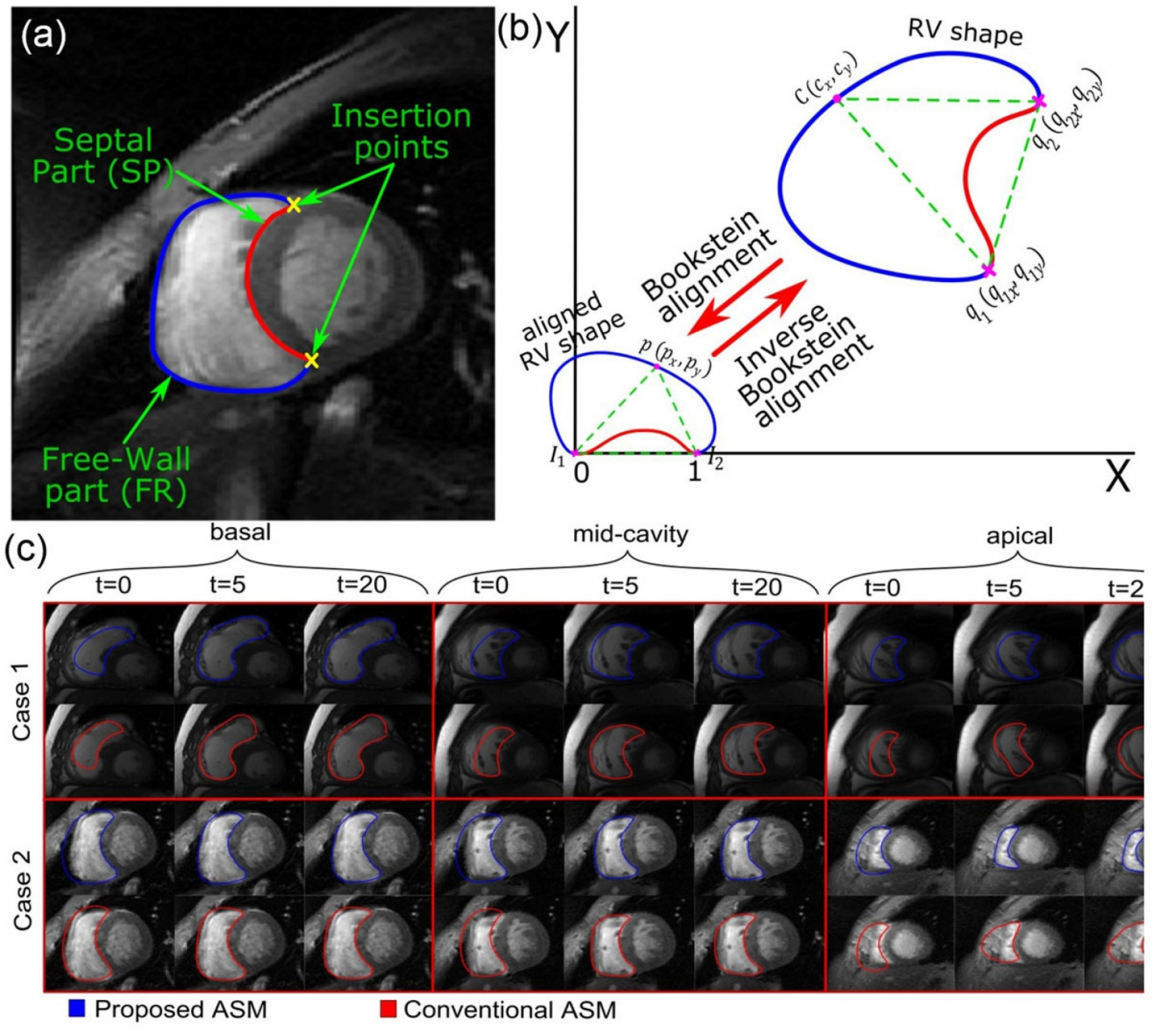
**(a) The RV contour is divided into 2 segments (septal and free wall)**. (b) The transformation of the RV shape to the Bookstein Coordinates is performed in 2 steps: 1) registering the two insertion points to points 0 and 1 on the x-axis; and 2) normalizing each point on the original RV shape with respect to the distance between the insertion points. (c) The RV segmentation results in 2 cases using the proposed and conventional ASM models at the initial, 5th, and 20th interations. The figure shows 3 cross-sections at basal, mid-cavity and apical levels.

